# Analysis of incidence of motor neuron disease in England 1998–2019: use of three linked datasets

**DOI:** 10.1080/21678421.2021.2016837

**Published:** 2022-02-01

**Authors:** Judith M. Burchardt, Xue W. Mei, Tom Ranger, Christopher J. McDermott, Aleksandar Radunovic, Carol Coupland, Julia Hippisley-Cox

**Affiliations:** 1 Nuffield Department of Primary Care Health Sciences, Oxford University, Oxford, England; 2 Institute for Translational Neuroscience, University of Sheffield, Sheffield, England; 3 Barts MND Centre, Barts Health NHS Trust, London, England, and; 4 School of Medicine, University of Nottingham, Nottingham, England

**Keywords:** Epidemiology, motor neuron disease, amyotrophic lateral sclerosis, incidence, England, QResearch

## Abstract

*Objective:* This study uses three linked datasets to provide an estimate of incidence of motor neuron disease (MND) in England from 1998 to 2019. Comparison is made to previous British studies. It examines age at diagnosis and ethnicity of those affected.

*Methods:* The literature was searched for studies of MND incidence in Great Britain from 1995 to date. The QResearch and linked Hospital Episode Statistics and Death register databases were searched from 1998 to 2019 for cases of MND, and incidence calculated from 16.8 million adults and 112 million adult years of data.

*Results:* We found 6437 adults with a diagnosis of MND giving an incidence of MND of 5.69/100,000 person years (95% CI 5.51–5.88); 6.57 (6.41–6.99) in men and 4.72 (4.49–4.97) in women when age-standardized to the 2011 UK population. The median age of diagnosis was 72 years. Peak incidence occurred in the 80–84 year age group in men and 75–79 in women. Age-standardized incidence was as high in Bangladeshi, Black Caribbean, Indian, other Asian and Pakistani people as in White people. Black African and Chinese people had a lower incidence.

*Conclusion:* The use of three linked national datasets captured 33% more people than a primary care dataset alone. Patients were older than in previous studies and rates were high in all ethnic groups studied except Black African and Chinese people. We present the highest incidence of MND reported globally in the past 50 years. Methodological differences may in part explain differences with previous reports. The use of national datasets may have captured additional MND patients with serious comorbidities who have not seen a neurologist before death. A limitation of this approach is that unlike population registers, which minimize false positive diagnosis by neurologist review of each patient, we cannot review diagnosis for individuals as data are anonymized.

## Introduction

Motor neuron disease (MND) is a progressive invariably fatal disease with a median life expectancy after diagnosis of only 1.5–3 years (1–4). MND is known as amyotrophic lateral sclerosis (ALS) when both upper and lower motor neurons are affected (2–4). ALS is the most common form of MND (2,3,5–8). Other less common forms of MND may progress to become ALS with time (3,5–7).

Neurologists have provided local estimates of the incidence of MND in England and a national register is being set up; crude incidences from these studies range from 1.06 to 2.98/100,000 (6,9–12). A different approach is to examine numbers of patients registered with a general practitioner (GP) with a code for MND in their records. When the Clinical Practice Research Datalink (CPRD) GP database was searched from 1990 to 2005 the estimated incidence of MND was 4.3/100,000 in men and 3.1/100,000 in women over the age of 20. The source for that study was the GP records of over 3 million patients (6). Using QResearch, a nationally representative database of English GP medical records, we have calculated updated estimates based on 16.8 million individuals of differing ethnicities from 1998 to 2019. The GP records of patients in QResearch are linked to their Hospital Episode Statistics (HES) and to the English death register databases. All three datasets were searched for this study.

## Methods

We used version 44 of QResearch, a large validated primary care electronic database containing anonymized medical codes for 16.8 million English patients registered with 1500 general practices using the Egton Medical Information System (EMIS) computer system (13).

The base cohort included individuals 18 years and over registered during the study period (1 January 1998–31 December 2019) without a diagnosis of MND at study entry. The study entry date was defined as the latest of 18th birthday; date of registration with the practice plus one year; date on which the practice computer system was installed plus one year; or the beginning of the study period (1 January 1998). The cohort was followed up until the earliest of the date of MND diagnosis; date of 100th birthday; date of death; date of leaving the practice; or the study end date (31 December 2019).

Cases were defined as patients in the study cohort with a new diagnosis of MND on the earliest of general practitioner (GP) record, Hospital Episode Statistics (HES) record or Office of National Statistics (ONS) death register during follow-up.

The QResearch database was searched for the Read codes, F152 MND, F1520 ALS, F1521 Progressive Muscular Atrophy, F1521-Duchenne Aran Muscular Dystrophy, F1522 Progressive Bulbar Palsy, F1523 Pseudobulbar Palsy, F1524 Primary Lateral Sclerosis and F152z MND not otherwise specified and the Hospital Episode Statistics and Death Register datasets for ICD 10 G12.2 codes MND. Data on year of birth, sex, and ethnicity were taken from GP records.

GPs add clinical codes to patients’ records both from their own diagnoses, and from clinic and discharge letters sent them by hospital colleagues. These letters are also independently coded for the hospital episode statistics (HES) database. In the UK, every person has a unique National Health Service (NHS) number. QResearch generates a pseudonymized version of the NHS number which is shared across the linked HES and death register databases. It is therefore impossible for any person with MND to be counted more than once.

### Literature review

PubMed was searched for studies of MND Incidence in the UK from January 1995 to October 2021 using the keywords Motor Neuron Disease, Incidence, England, Wales, Scotland, Britain, United Kingdom. Studies were reviewed on title and abstract by J. B. for those including estimates of MND incidence in Great Britain.

### Statistical analyses

Summary statistics were generated for age, sex and ethnicity. The median (interquartile (IQR)) age was also calculated stratified by source of diagnosis. Crude incidence rates were calculated as the number of MND events divided by person-time, and by categories of age group, self-reported ethnicity, and calendar year. Age-standardized rates were calculated using the direct method against the UK 2011 census and US 2010 population and presented overall and stratified by sex (14,15). Incidence rates were further stratified by source of MND diagnosis (i.e. any source, GP and/or HES record, or death register only). Age and sex standardized incidence rates are also presented by ethnicity. 95% Confidence intervals for crude rates were calculated using the quadratic approximation to the Poisson log likelihood for the log-rate parameter.

## Results

### Literature review

Of the 313 studies identified in the search, 8 provided estimates of either crude and/or age-standardized MND incidence in England, Wales, or Scotland (collectively known as Great Britain) (5–7,9–12,16) (Table 1).

**Table 1:** Incidence rates of MND from Qresearch compared to previous studies of MND in Great Britain.

Study	Setting	Study period	N incidence	Sample size	Age	Crude incidence rate /100 000 Person Age Years (95% confidence intervals)	Age-standardized to UK 2011 population all ages incidence rate /100 000 person years (95% confidence intervals)	Age-standardized to US 2010 population all ages incidence rate /100,000 person years (95% confidence intervals)
QResearch (this study)	England (GP, HES, or death)	1998–2019	6437	16,799,922 (112,003,453 patient years)	18+	5.75 (5.61–5.89)	5.69 (5.51–5.88)	5.02 (4.95–5.10)
Opie-Martin et al. (2020) (11)	England (Six MND registers including imputed cases)	2017–2018	287–301	7,251,845	16+	–	2.072 (2.072–2.073)	1.874 (1.873–1.874)
Leighton et al (2019) (5)	Scottish MND Register	2015–2016	406	5,388,850	16+	3.83 (3.53–4.14)	–	3.42 (2.99–3.9) in 2015 2.89 (2.50–3.34) in 2016
Gowland et al (2019) (12)	South London and Canterbury (SEALS register)	2004–2017	415	–	15+	2.27 (2.04–2.50)	2.74 (2.59–2.88)^b^	–
Imam et al (2010) (9)	Devon and Cornwall, England (hospital register)	2002–2007	243	–	15+	2.98	–	–
Alonso et al (2009) (7)	England^a^ (GP data from CPRD database)	1990–2005	830	>3,000,000 (22,492,571 person years)	20+	3.69 (3.44–3.95)	–	–
Abhinav et al (2007) (6)	South East England (ALS Register)	2002–2006	138	2,890,482	15+	1.06	–	–
Forbes et al. (2007) (16)	Scottish MND Register	1989–1998	1226	5,1215,000	10+	2.38 (2.25–2.52)	–	–
Mitchell et al. (1998) (10)	Lancashire and South Cumbria, England (neurologist knowledge)	1989–1993	130	1,473,153	NS	1.76	–	–

^a^Although this paper is reported as being from the UK, the CPRD (then GPRD) database from which it was taken was only linked to English practices at the time. Crude rate and confidence intervals were calculated from data given in the paper.

^b^2010 UK population. NS: not specified.

### Results from QResearch, HES and death registry database search

We found 6437 cases in 112,000,000 adult years giving a crude incidence of 5.75 (95% CI 5.61–5.89)/100 000 adult years. Of these, 5773 (90%) cases were recorded in HES. By using HES and death register data 33% more MND cases were found that would have been had GP data alone been used. No patients were coded by their GPs unless they also had a HES code. Six hundred sixty-four (10%) patients were recorded on death certificates alone (Figure 1). The median age of diagnosis was 72 (IQR 64–80) years. After excluding cases found only in the death register the median remained 72 years (IQR 63–79). Only 19% of cases were found on a single dataset (Table 2).

**Table 2:** Crude and age-standardized incidence of MND (95% confidence intervals) and median age by data source.

	All MND patients (including death register) (*n* = 6437)	Male MND patients (including death register) (*n* = 3656)	Female MND patients (including death register) (*n* = 2781)
Crude incidence adult MND/100,000 adult years	5.75 (5.61–5.89)	6.61 (6.40–6.83)	4.91 (4.72–5.09)
UK 2011 age-standardized incidence/100,000 person years using all 3 datasets	5.69 (5.51–5.88)	6.69 (6.41–6.99)	4.72 (4.49–4.97)
US 2010 age-standardized Incidence/100,000 person years using all 3 datasets	5.02 (4.95–5.10)	5.82 (5.70–5.94)	4.26 (4.16–4.36)
Median age of diagnosis in years (interquartile range)	72 (64–80)	72 (63–79)	73 (65–80)
	MND patients in HES and QResearch (*n* = 5773)	Male MND patients in HES and QResearch (*n* = 3271)	Female MND patients in HES and QResearch (*n* = 2502)
UK 2011 age-standardized Incidence/100,000 person years excluding death registry patients	5.23 (5.05–5.41)	6.01 (5.74–6.29)	4.47 (4.24–4.71)
Median age of diagnosis in years (interquartile range)	72 (63–79)	71 (63–78)	73 (65–80)

MND incidence rose over the time period of the study (Figure 2). MND was found to be more common in men than women with a sex ratio of 1.5:1. Incidence rates peaked in the 80–84 year age group for men and the 75–79 year age group for women when all cases were included (Figure 3). The median age of patients at diagnosis was 72 (IQR 64–80) years. For men, the median age of diagnosis was 72 (IQR 63–79) and for women 73 (IQR 65–80) years. When patients identified only on the death register were excluded from the analysis the peak age group for MND incidence was 75–79 years for both men and women with a median age of 72 (IQR 63–79) years.

Age-standardized incidence for MND was similar in Bangladeshi, Caribbean, Indian, other, other Asian, Pakistani and White people and relatively lower in Black African and Chinese people (Figure 4).

## Discussion

### High incidence of MND in England

Our study found England to have a higher incidence of MND than any other country with published data at 5.69 (5.51–5.88) per 100,000 person years when age-standardized to the UK 2011 census and 5.02 (4.95–5.10) when standardized to the US 2010 population. The next highest incidence rates of MND reported globally in recent times are also from Great Britain—an estimated 3.2 (3.0–3.5)/100,000 person years in England in 2009 standardized to the 2000 US population and 3.42 (2.99–3.91) and 2.89 (2.50–3.34)/100,000 in Scotland in 2015 and 2016, respectively, to the 2010 US population (5,7). A 2012 study of the French population found a lower rate of 2.72/100,000 person years after age-standardization to the 2010 US population (17). Possible reasons for our high reported incidence of MND in England will be discussed in turn.

#### Higher crude incidence with an aging population

We have more complete data capture than previous UK studies because we used three national datasets (6,7,9,11,18). Had we used data from GP records alone we would have found a crude MND incidence of 4.29 (95% CI 4.17–4.42)/100,000 in 18+ year olds similar to the 3.69 (3.44–3.95)/100,000 person years found in 20+ year olds when the CPRD database was searched from 1990 to 2005 (7). By adding hospital and death register data, the crude incidence increased by 33% to 5.75/100,000 adult years. Furthermore, our results are comparable to those of the Million Women study in 2012 which also used HES and death register data from the UK, and identified 8% of their 752 cases on the death register only, as compared to 10% of ours (18). They found an incidence of 6.08 (5.65–6.54)/100,000 woman years compared to ours of 10.36 (9.89–10.85)/100,000 in women aged 50–74. We have found a much higher incidence than that from the England, Northern Ireland, and Wales population-based register (11). Patients counted in population based MND registries are all reviewed by specialist neurologists, but even so some will be misdiagnosed with MND, and later have the diagnosis revoked (19). In addition to this over-reporting of MND incidence, under-reporting may also occur; population registers cannot include those who are not seen by a neurologist, a particular risk for elderly patients with multi-morbidity (20,21). The older patients and higher incidence found in our study may be due to elderly patients who are not referred, or die without seeing a neurologist, and are therefore not included in population registers.

##### Higher crude incidence with an aging population

Diagnosis of MND became more common over the course of the study period, in keeping with a finding of the Global Burden of Disease study which found a 34.5% increase in the prevalence of MND and spinal muscular atrophy in the UK from 1990 to 2016 (22). This is likely to be another reason that our crude incidence figures were higher than previous studies; our study overlapped with that from the CPRD for only 7 years, and rates have increased since then.

Between 1998 and 2017 life expectancy in England rose from 75 to 80 years in men and from 80 to 83 years in women (23). This will have increased the crude incidence of MND, given its strong association with age (24). An international meta-analysis of studies of ALS since 1885 found an increase in its crude, but not age-standardized incidence (25).

##### Increasing case ascertainment

As in our study, MND registries in Scotland, Northern Italy, Sweden, and Denmark have also reported an increase in its age-standardized incidence (5,26–29). An increased recognition of phenotypes such as those in the El Escorial “possible ALS” group may be an important cause of this increase (29). The number of neurologists in the UK has increased over the period of this study, from 0.43/100,000 in 1995 (30) to 0.8/100,000 in 2011 (31) and 1.1/100,000 currently (32). In addition, the profile of MND has been raised by the work of the MND Association, inaugurated in 1979, which now supports sixteen MND care centers and six networks in England, Wales and Northern Ireland. This is likely to have increased both patients’ and clinicians’ awareness of MND and access to neurologists, and hence the likelihood of people receiving a diagnosis of MND.

### Median and peak age of MND incidence

Previous studies from Europe have found a median age of diagnosis of ALS of 65 years for men and 67 for women (range 63–9) years and from South East England a median of 61 years (6,11,16,17,26,28,33,34). The median age of 72 years at diagnosis in our study is considerably older. This may reflect better case finding, but we also considered that the 664 cases (10%) who were only coded at death could be elevating the figures. The data were therefore re-analyzed excluding cases found only on the death register; the peak age of study entry for MND cases became 75–79 for both men and women. The median age remained 72 years. Median age at diagnosis of MND in the death register only patients was 77 (IQR 71–83) years.

Many national studies and a worldwide meta-analysis have found that incidence decreases after the age of 80 (11,16,17,26,33,35). A Swedish study found male incidence of MND peaked over the range of 70–84 years of age (27). Our finding of peak age of MND incidence of 75–9 in women is in line with previous studies, but the peak age of incidence of 80–84 in men in our study is, to our knowledge, the highest ever reported.

Our study captured older patients than those identified in studies from secondary and tertiary referral centers (6,11,26,33,36). The use of real world evidence may have identified patients with MND who have not been referred to a neurologist. It is conceivable that their GP or hospital doctor made a clinical diagnosis of MND but did not code it as such in the absence of neurological confirmation, but then gave it as their clinical opinion of the cause of death. Therefore, recording bias may have given spuriously low findings for incidence studies not using death register data. We cannot tell if the increase in age-standardized incidence in our study is due to a true increase in the disease, incorrect diagnosis of MND, or better case ascertainment.

### Scope: MND, not ALS alone

Many other studies have examined the incidence of ALS which makes up 67–95% of MND cases (5–7,11,12,27). We included patients with all forms of MND in our study. Patients with ALS are coded as having MND in the UK, so we were unable to study ALS incidence alone.

Studies of ALS incidence show a similar pattern to those of MND, with higher incidence in men than women and increasing incidence with age until at least 75 years, albeit with lower figures, as ALS is only one type of MND. Meta-analyses of global incidence of ALS have found a crude rate of 1.59–1.75/100,000 with heterogeneity such that incidence in Northern Europe at 1.89/100,000 is more than twice as high as that in Asia (25). A previous systematic review found the median incidence rate of ALS in Europe to be 2.08/100,000 (37). A recent study of ALS in England estimated an incidence of 2.07/100,000 (2.07–2.08) based on 232 cases diagnosed from 2017 to 2018 in six MND centers covering 13% of the population of England (11). ALS incidence in Sweden is 2.97/100,000, and this also had increased considerably over time (27).

### MND more common in men

In our study, MND diagnosis was more common in men with a male:female ratio of 1.5:1. The higher incidence in men has been found in many previous studies (6,7,12,16,17,25,28,34–36,38).

### Age-standardized incidence of MND similar in all ethnic groups except black Africans and Chinese people, who had lower incidence

Crude incidence of MND was 6.05/100,000 adult years in White people, which was higher than in any other ethnic group (data not shown). Age-standardized ethnicity was similar for Bangladeshi, Caribbean, Indian, Pakistani, other, other Asian, and White people. It was less common in Black African and Chinese people. Numbers were low in all groups except White. Our findings are similar to those of a Cuban study which found similar rates of ALS in Caribbean and White people and a worldwide meta-analysis which confirmed this and also found lower rates in Chinese people (25,39). The relatively lower age-standardized incidence of ALS in Black African and Chinese people has also been found in recent studies of 430 million urban Chinese people and Black as compared to White South African people (35,38). To our knowledge, there is no previously published data on MND incidence in people from the Indian subcontinent.

Self-reported ethnicity was not recorded for 45% of MND cases and 29% of controls matched for age, sex, GP practice, and date of record. However, when only cases and controls with reported ethnicity were analyzed, ethnicity was very similar in the two groups (Mei et al. Identification of key red flag signs and symptoms of motor neuron disease in primary care: a nested case–control study using the Qresearch database—unpublished.)

### Strengths and limitations

This is the largest ever study of MND incidence in England with a sample size of 16.8 million people and 6437 cases identified. We used real-world evidence from three linked datasets over 23 years and included all forms of the disease.

It is not possible to individually confirm the diagnosis of MND for each patient as our data is anonymized. Potential inaccuracy of coding is a significant limitation of our study: it seems likely that not all patients recorded by HES and the death register were assessed by a neurologist. Several studies have cast doubt on the accuracy of death certificate diagnoses of MND (40–42). However, it is also recognized that use of linked GP records is a powerful method for maximizing case ascertainment (43,44). A systematic review of the accuracy of routinely collected healthcare data for MND found positive predictive values of 55–92% and sensitivities of 75–93%, with UK studies found to be the most accurate (45). When 81 GP recorded MND diagnoses were reviewed by a neurologist in the CPRD study, the diagnosis was confirmed in 85% of cases (7). Subsequent studies have confirmed the reliability of coding of MND (46) and in the current study 81% of the patients were identified by at least two data sources, which supports the reliability of identifying cases in this way.

## Conclusion

This is the largest study yet undertaken of the incidence of MND in England, based on the records of 16.8 million patients linked through three national datasets. We found an age-standardized incidence of 5.69/100,000 person years, much higher than the rate reported in population registers. The median age of diagnosis was 72, significantly older than in other studies. This high age-standardized incidence was found in Bangladeshi, Black Caribbean, Indian, other Asian, Pakistani, and White people. Black African and Chinese people had a lower incidence. One explanation for the high incidence of MND compared to that reported in other studies is that MND may be falsely diagnosed on death registry or hospital episode statistics by non-neurologists. However, further research should also consider the possibility that some elderly multi-morbid patients with MND and other life-limiting conditions may not be referred to neurologists, and hence escape traditional case ascertainment methods.

## Data Availability

The study protocol and statistical analysis plan for this project are available on request from the corresponding author. De-identified individual participant data that underlie the results reported in this article will be made available, with requests accepted immediately after publication, for proposals that set out to achieve aims specified in a methodologically and scientifically sound protocol that are approved by the Qresearch Scientific Advisory Committee (“learned intermediary“), where costs of providing access to the data are covered, where requests are compliant with the legal permissions of Qresearch data providers, and Qresearch data security requirements are met. Information regarding submission of applications to access data can be found online: www.qresearch.org/. Data sources for MND cases – GP records, Hospital Episode Statistics and Death Certificates 1998–2019. Crude MND Incidence/100,000 adult years in England 1998–2019. Crude incidence by age at study entry for MND/100,000 adult years by age and sex in England 1998–2019. Age-standardized incidence MND/100,000 person years in England by self-reported ethnicity 1998–2019.
